# Tinkering the Postal Laws

**Published:** 1881-12

**Authors:** 


### TINKERING THE POSTAL LAWS.

SEVERAL years ago the law permitting merchandise in packages of not more than four pounds each to be sent through the mails at the rate of one-half cent per ounce was repealed, as stated at the time, in the interest and through the connivance of certain express companies.

The people demanded that the law should be restored, and it was restored, but the rate was doubled; we thus pay one cent per ounce, which is 16 cents per pound; this is at the rate of $320.00 a ton. If we add the fractions of ounces that are paid for as full ounces, and the 10 cents each for such packages as are "registered," it will be found that the government receives about $400.00 per ton for all merchandise that passes through the mails; which is just *ten* times as much as is charged for second-class matter. Yet, Postmaster-General James, in his report, just published, says: "That the business of carrying merchandise in the mails is steadily unremunerative, and the law permitting it should be repealed." Funny! ain't it?

